# Supplementary material for: Bacterial survival in microscopic surface wetness
Source: eLife. 2019 Oct 15;8:e48508. doi: 10.7554/eLife.48508 (PMC6824842; doi:10.7554/eLife.48508)
Supplement: Supplementary file 1. — Calculated molarity and osmolarity of salt components in M9 medium at the standard concentration (M9 1x) and in estimated concentrations within microdroplets (M9 23.3x). [file elife-48508-supp1.docx]

|  | **M9 1x** | | **M9 23.3x** | |
| --- | --- | --- | --- | --- |
| **Salt** | **Molar concentration**  **[mM]** | **Osmolarity**  **[mOsm/L]** | **Molar concentration**  **[mM]** | **Osmolarity**  **[mOsm/L]** |
| Na_2_HPO_4_ | 47.8 | 143.3 | 1113.7 | 3338.89 |
| KH_2_PO_4_ | 22.0 | 88.2 | 512.6 | 2055.06 |
| NaCl | 8.6 | 17.1 | 200.4 | 398.43 |
| NH_4_Cl | 18.7 | 37.4 | 435.7 | 871.42 |
| **Total** |  | **286.0** |  | **6663.8** |

**Supplementary Table 1. Molar concentration and osmolarity of M9 salts.** Calculated molarity and osmolarity of salt components in M9 medium at the standard concentration (M9 1x) and in estimated concentrations within microdroplets (M9 23.3x).
