# Supplementary material for: Bacterial survival in microscopic surface wetness
Source: eLife. 2019 Oct 15;8:e48508. doi: 10.7554/eLife.48508 (PMC6824842; doi:10.7554/eLife.48508)
Supplement: Supplementary file 2. — Plate Reader (Synergy H1, BioTek) screen results were analyzed using GrowthRate and GRplot programs (Mira, P., M. Barlow, and B. G. Hall. Statistical Package for Growth Rates Made Easy. Mol. Biol. Evol. 34:3303–3309, 2017). Results of zero growth were omitted from this table. In both strains, the general picture was that higher salt concentrations led to a decrease in growth rate, a decrease in final OD, and an increase in lag time. ‘*’: R is lower than 0.99. [file elife-48508-supp2.docx]

| Strain &  growth conditions | Growth Rate  [hr^-1^] | R | Max OD | Doubling time [minutes]  (Mean±SD) | Lag time [hours] |
| --- | --- | --- | --- | --- | --- |
| A506 M9-salts 0.5x | 0.46 | 0.9994 | 0.2812 | 90 ± 2 | 3 |
| A506 M9-salts 1x | 0.54 | 0.9962 | 0.3049 | 78 ± 4 | 3 |
| A506 M9-salts 5x | 0.49 | 0.9922 | 0.4234 | 85 ± 7 | 10 |
| A506 M9-salts 10x | 0.03 | 0.9934 | 0.0542 | 1490 ± 81 | 1 |
| A506 M9-salts 20x | NA | NA | 0.0339 | NA | NA |
| A506 NaCl 10mM | 0.60 | 0.9906 | 0.2616 | 69 ± 6 | 5 |
| A506 NaCl 100mM | 0.43 | 0.9967 | 0.4216 | 96 ± 4 | 5 |
| A506 NaCl 500mM | 0.30 | 0.9985 | 0.4572 | 139 ± 4 | 17 |
| A506 NaCl 1000mM | 0.10 | 0.9991 | 0.0649 | 403 ± 9 | 16 |
| A506 NaCl 2000mM | NA | NA | 0.0276 | NA | NA |
| KT2440 M9-salts 0.5x | 1.16 | 0.9838* | 0.2826 | 36 ± 4 | 4 |
| KT2440 M9-salts 1x | 0.90 | 0.9891* | 0.1859 | 47 ± 4 | 4 |
| KT2440 M9-salts 5x | 0.27 | 0.9978 | 0.2672 | 154 ± 5 | 6 |
| KT2440 M9-salts 10x | 0.20 | 0.9993 | 0.3689 | 210 ± 4 | 26 |
| KT2440 M9-salts 20x | NA | NA | 0.0472 | NA | NA |
| KT2440 NaCl 10mM | 0.53 | 0.9993 | 0.1849 | 79 ± 2 | 5 |
| KT2440 NaCl 100mM | 0.52 | 0.9970 | 0.1902 | 80 ± 4 | 5 |
| KT2440 NaCl 500mM | 0.23 | 0.9995 | 0.3102 | 180 ± 3 | 10 |
| KT2440 NaCl 1000mM | 0.14 | 0.9985 | 0.2089 | 311 ± 7 | 46 |
| KT2440 NaCl 2000mM | NA | NA | 0.0372 | NA | NA |

**Supplementary Table 2. Growth curve analysis of *P. fluorescens* A506 and *P. putida* KT2440 at different M9 concentrations and NaCl concentrations.** Plate Reader (Synergy™ H1, BioTek™) screen results were analyzed using GrowthRate and GRplot programs (Mira, P., M. Barlow, and B. G. Hall. Statistical Package for Growth Rates Made Easy. Mol. Biol. Evol. 34:3303-3309, 2017). Results of zero growth were omitted from this table. In both strains, the general picture was that higher salt concentrations led to a decrease in growth rate, a decrease in final OD, and an increase in lag time. ‘*’: R is lower than 0.99.
